# Potential predictors for progression of moyamoya disease: A systematic review and meta-analysis

**DOI:** 10.3389/fneur.2023.1128338

**Published:** 2023-03-02

**Authors:** Jun Cao, Zixuan Xing, Ling Dai, Tao Wang, Yuhai Zhang, Yao Feng, Yanfei Chen

**Affiliations:** ^1^Department of Neurosurgery, Xuanwu Hospital, Capital Medical University, Beijing, China; ^2^Department of Neurosurgery, The Affiliated Rizhao People's Hospital, Jining Medical University, Rizhao, China; ^3^Health Science Center, Xi'an Jiaotong University, Xi'an, China; ^4^Department of Neurosurgery, Jinshan Hospital, Fudan University, Shanghai, China

**Keywords:** moyamoya disease, potential predictors, progression, systematic review, meta-analysis

## Abstract

**Background:**

The progress of Moyamoya disease (MMD) is often accompanied by the occurrence of new ischemia or hemorrhagic events, which was difficult to predict. This systematic review and meta-analysis aimed to identify predictors for progression in MMD patients.

**Methods:**

We searched PubMed, Web of Science, Cochrane Library, and Embase databases up to December 10th, 2022 for randomized controlled trials, case-control studies, or cohort studies reporting predictors of disease progression in MMD patients. The results of each predictor were pooled by meta-analysis and further analyzed by subgroup analysis for predictors of unilateral to bilateral progression of MMD.

**Results:**

A total of 842 patients from 12 studies were included. The estimated pooled means indicated lower age (standard mean difference [SMD]: −0.29, 95% confidence interval [CI]: −0.55 to −0.03; *P* = 0.03), family history (odds ratio [OR] 3.97, 95% CI: 1.96 to 8.03; *P* < 0.001) and contralateral abnormality (OR 3.95, 95% CI: 1.10 to 14.20; *P* = 0.04) were associated with progression in MMD patients. Subgroup analyses indicated that the same three factors were associated with the progression of unilateral to bilateral MMD.

**Conclusions:**

This meta-analysis revealed that lower age, family history and contralateral abnormality were associated with progression in MMD patients. The same three factors are associated with the progression of unilateral to bilateral MMD. Further studies are needed to validate our results.

## Introduction

Moyamoya disease (MMD) is a chronic cerebrovascular disease characterized by progressive narrowing of the distal internal carotid arteries (ICA) and their proximal branches, finally developing abnormal collateral vessels, including basal moyamoya vessels, leptomeningeal anastomosis, and transdural anastomosis(1, 2). MMD is more common in people living in East Asian countries such as Japan (3–6) and Korea (7, 8) than in the Western Hemisphere (9, 10). In Japan, the incidence rate is 0.94–1.13 per 100,000 people, and the prevalence rate is 5.22–10.50 per 100,000 people (4, 6). Cognitive function and physical dysfunction caused by MMD seriously affect the quality of life of patients (1, 11). With the development and application of radiological techniques, various neuroimaging methods with different advantages have furthered the understanding of MMD in terms of its structural, functional, spatial, and temporal aspects. Such angiographic and morphological changes of this chronically progressive disease have been gradually understood, but the mechanisms of the disease progression have not been fully elucidated.

Disease progression may be related to the development of new ischemic or hemorrhagic events (12). For patients with MMD, the mortality rate of the first hemorrhage is 6.8%. Once the hemorrhage occurs again, the mortality rate will rise sharply to 28.6% (13). Predicting disease progression may reverse the current passive situation of waiting for hemorrhage before treatment. At the same time, it can assist clinical decision support and help patients clearly understand their own disease status, thereby indirectly improving their quality of life during treatment.

Some studies have explored the factors that can predict the progression of MMD, but there are also some controversies between them. Contralateral abnormality, age, family history and so on were found to be different between progression and non-progression groups in previous studies (2, 14, 15). However, Tian et al. (16) didn't find any predictors to distinguish these two groups. As most related studies are observational ones with a low level of evidence, a systematic review and meta-analysis to determine predictors of progression in MMD patients was necessary.

## Methods

### Eligibility criteria

The inclusion criteria were as following:

1. Patients: Patients were diagnosed with MMD which Refer to the diagnostic criteria revised in 2021 (17).
2. Potential predictors: Include at least one of the following potential predictors: age, sex, initial lesion side, Suzuki grade, hypertension, diabetes, family history, smoking and contralateral abnormality.
3. Outcome: After at least 6 months of follow-up, the disease was found to have progressed, which meant progression of the occlusive lesion in the major intracranial arteries or from unilateral to bilateral lesions.
4. Study type: Randomized controlled trials, case-control studies or cohort studies.
5. Publication date and language: Limited to articles published on or before December 10th 2022, English.

Studies were excluded based on the following criteria:

1. Patients: Moyamoya syndrome, atherosclerotic lesion and inflammatory disease.
2. Potential predictors: None of the above potential predictors were reported.
3. Study type: Case reports, expert consensus, abstracts, conferences, animal studies, guidelines, and comments.
4. Language: Non-English.

### Search strategy

RCTs, case-control and cohort studies reporting progression of MMD patients were included after searching PubMed, Web of science, Cochrane library and Embase databases. There were no restrictions on the type of study used, and the publication date and language filters corresponding to the eligible criteria were used. The following search terms were used in different combinations: moyamoya disease; moyamoya syndrome; disease progression; disease exacerbation; progression.

### Study selection and data extraction

Two authors (JC and ZX) independently screened and selected the eligible studies according to the inclusion and exclusion criteria. Data were independently extracted by 2 authors (JC and ZX) from all the included studies and subsequently cross-checked to ensure their accuracy. Any discrepancies in study extraction and data extraction were resolved by the senior author during cross-checking process. Data extracts included the first author of the study, year of publication, definition of progression, quality assessment, recruitment period, follow-up duration and characteristics of the study population in total, including the number of participants with potential predictors like age, sex, Suzuki grade, comorbidities et al. which were available in the literature. Authors were contacted for missing information when necessary. Studies were excluded when there was no response upon exposure.

### Assessment of risk of bias

Two authors (JC and ZX) independently assessed the risk of bias for included studies according to the principle of the Newcastle-Ottawa Scale (NOS) (18). Potential predictors that were included in <10 studies were not suitable for performing funnel plots because of the potential difficulty in obtaining sufficient power to distinguish random from genuine occurrence (19). Any disagreements were resolved by the 2 authors through discussion, with the help of a third author (LD).

### Assessment of heterogeneity

The I-square test was used to test the heterogeneity. We defined heterogeneity as follows: I^2^ = 25–49%, low heterogeneity; I^2^ = 50–74%, moderate heterogeneity; and I^2^ > 75%, severe heterogeneity.

### Data synthesis and statistical analysis

Continuous outcomes were presented as standardized mean difference (SMD) with 95% confidence interval (CI), and dichotomous outcomes were described as odds ratio (OR) with 95% CI. A meta-analysis was conducted using the software Review Manager if the effect sizes were available or calculable in 3 or more studies for specific potential predictors. A random-effects model was used to analyze the outcomes for included studies, but a fixed-effect model was used when there was little evidence of heterogeneity (I^2^ < 50%). Moreover, Subgroup analysis would be performed according to the content of included studies.

## Results

### Study selection and study characteristics

**Figure 1:**
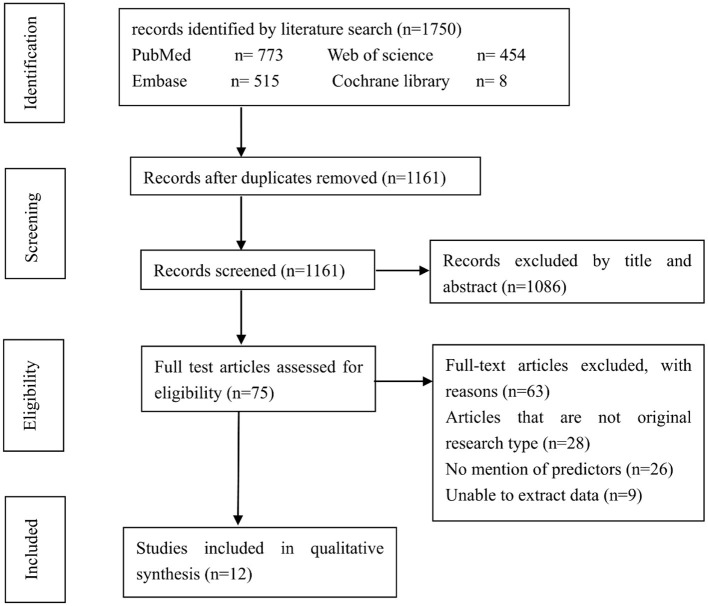
Flow chart of the study selection.

There were 1,750 potentially relevant studies being systematically identified through an electronic database search, 589 of which were excluded due to duplication. Further, 1,086 studies were excluded after a screening of the titles and abstracts. Another 63 studies were excluded after reviewing the full text. These studies were excluded because they were not original research, didn't mention potential predictors or unable to extract data. Finally, 12 studies enrolling a total of 842 patients (158 patients with progression and 684 patients with non-progression) were included in the meta-analysis (2, 12, 14–16, 20–26). The flowchart for studies screening was presented in Figure 1.

The included studies had been published between 1994 and 2022. There were no randomized controlled trials. The mean incidence of MMD progression of the included studies was 18.8% (range 8.2–58.8%). Most of studies focused on the progression from unilateral to bilateral lesions. The detailed characteristics of the 11 included studies were listed in Table 1.

**Table 1:** Characteristics of included studies for progression of MMD.

**Studies**	**Country**	**Period**	**Definition of progression**	**Follow-up duration**	**Patients**	**Total patients**	**Rate of progression**	**Factors included in analysis**	**Quality (NOS)**
Kawano et al. (26)	Japan	NA	From unilateral to bilateral lesions	Progression: 0.7–7 (mean 2.6) years Non-progression: 0.2–13 (mean 3.1) years	Children and adults	32	53.1%	Age, sex, family history	5
Kuroda et al. (12)	Japan	1990–2004	progression of the occlusive lesion in the major intracranial arteries	73.6 ± 49.0 months	Adults only	63	23.8%	Age, sex	6
Kelly et al. (20)	USA	January 1, 1991–December 31, 2005	From unilateral to bilateral lesions	Progression: 17.1 ± 3.5 months Non-progression: 19.0 ± 3.3 months	Children and adults	18	38.9%	Sex, hypertension, diabetes, contralateral abnormality	6
Smith and Scott (21)	USA	January 1, 1985–June 30, 2006	From unilateral to bilateral lesions	5.3 years (first operation) and 4.3 years (second operation)	Children and adults	33	30.3%	Family history	5
Park et al. (14)	South Korea	January 2000–June 2008	From unilateral to bilateral lesions	18.1–100 (mean 35.3) months	Children only	34	58.8%	Age, sex, family history	7
Yeon et al. (22)	South Korea	March 1995–February 2009	From unilateral to bilateral lesions	13–157 (mean 53.4) months	Children only	45	17.8%	Sex, initial lesion side, Suzuki grade	7
Lee et al. (15)	South Korea	2001–2011	From unilateral to bilateral lesions	50.1 ± 28.0 months	Adults only	41	14.6%	Age, sex, hypertension, family history, smoking, contralateral abnormality	7
Zhang et al. (23)	China	January 2002–May 2014	From unilateral to bilateral lesions	43.8 ± 21.3 months	Children and adults	109	16.5%	Sex, initial lesion side, Suzuki grade, hypertension, diabetes, smoking, contralateral abnormality	7
Church et al. (25)	USA	1991–2017	From unilateral to bilateral lesions	1–22 (average 5.8) years	Adults	217	8.3%	Sex, hypertension, family history, smoking	7
Mineharu et al. (2)	Japan	1987–2017	From unilateral to bilateral lesions	0–355 (mean 72.2) months	Children and adults	93	24.7%	Age, sex, hypertension, diabetes, family history, smoking, contralateral abnormality	7
Oomori et al. (24)	Japan	May 2008–September 2015	Scores in one or more main cerebral arteries were increased	5 years	Adults only	68	11.8%	Age, sex, hypertension, diabetes	8
Tian et al. (16)	China	January 2015–January 2017	From unilateral to bilateral lesions	48–78 (median 43) months	Adults only	89	8.2%	Sex, initial lesion side, Suzuki grade, hypertension, diabetes, family history	7

MMD, moyamoya disease; NOS, Newcastle-Ottawa Scale; NA, not available.

### Risk of bias of included studies

The Newcastle–Ottawa scale was used to assess the bias risk of observational studies (Table 1). All the included studies were assessed with 5 to 9 points by NOS. Funnel plot was unsuitable for assessment of publication bias because the number of included studies for analyzing potential predictors were small.

### Meta-analysis

**Figure 2:**
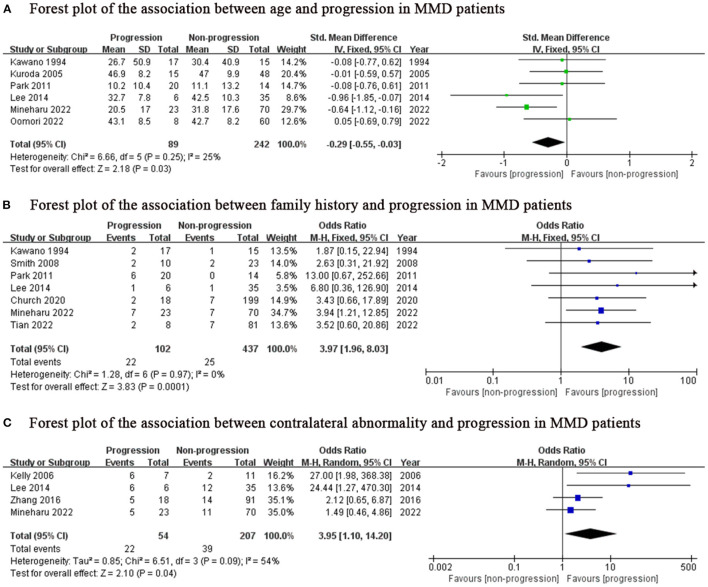
Positive predictor for progression in MMD patients. MMD, moyamoya disease; SD, standard deviation; IV, Inverse variance; M-H, Mantel-Haenszel; df, degrees of freedom; CI, Confidence Interval; Std. Mean Difference, standardized mean difference.

**Figure 3:**
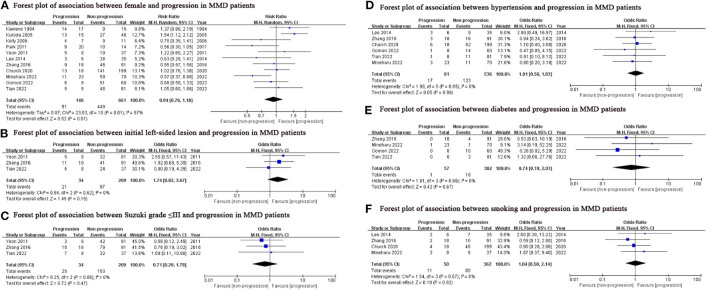
Potential predictors which were not significantly associated with the progression in this meta-analysis. MMD, moyamoya disease; M-H, Mantel-Haenszel; df, degrees of freedom; CI, Confidence Interval.

We found lower age (SMD: −0.29, 95% CI: −0.55-−0.03; *P* = 0.03), family history (OR: 3.97, 95% CI: 1.96–8.03; *P* < 0.001) and contralateral abnormality (OR: 3.95, 95% CI: 1.10–14.20; *P* = 0.04) were associated with progression in MMD patients (Figure 2), while sex, initial lesion side, Suzuki grade, hypertension, diabetes and smoking were not (Figure 3).

### Subgroup analysis

**Figure 4:**
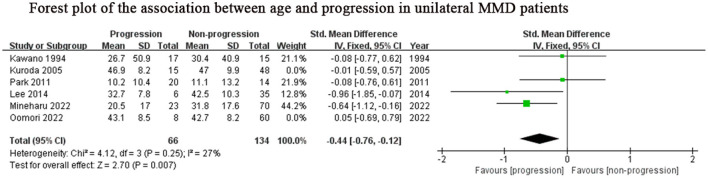
Positive predictor for progression in unilateral MMD patients. MMD, moyamoya disease; SD, standard deviation; IV, Inverse variance; M-H, Mantel-Haenszel; df, degrees of freedom; CI, Confidence Interval; Std. Mean Difference, standardized mean difference.

Considering the inclusion of an “age” factor in two studies (12, 24) and the definition of progression in these two studies differently from the other studies (12, 24), we further performed a subgroup analysis of the remaining studies which focused on unilateral MMD patients. Same results as above, lower age (SMD: −0.44, 95% CI: −0.76–0.12; *P* = 0.007) was the predictor for progression of this subgroup (Figure 4). The results of family history (OR: 3.97, 95% CI: 1.96–8.03; *P* < 0.001) and contralateral abnormality (OR: 3.95, 95% CI: 1.10–14.20; *P* = 0.04) were consistent with those of the previous analysis due to consistent definitions about progression in unilateral MMD patients (Figures 2B, C).

## Discussion

Predicting the progression of the disease can achieve timely warning, early intervention, and reduce the disability and mortality of the disease, but the factors that predict the progression of MMD were still controversial. In this systematic review and meta-analysis of clinical studies reporting predictors of disease progression in MMD patients, a total of 842 patients were included, and we found three important findings: (1) the progression rate for MMD patients was around 18.8%; (2) lower age, family history and contralateral abnormality were associated with progression in MMD patients, while sex, initial lesion side, Suzuki grade, hypertension, diabetes and smoking were not predictors for progression; (3) lower age, family history and contralateral abnormality were associated with progression in unilateral MMD patients.

With the development and application of radiological techniques, more and more MMD patients are being identified (27). The incidence of progression in MMD patients was reported differently in these included literatures, varying from 8.2 to 58.8%. However, as for included literature, it seems that the newer the literature, the lower the incidence of progression, which may be due to the early prediction of disease recurrence, death, disability, and complications. The progression of the disease is often accompanied by the onset of symptoms, with more serious consequences for the patient (23). Identifying the predictors can inform the future progress of individual diseases and the probability of a certain outcome, so as to guide doctors and patients to jointly decide on future prevention, treatment, and rehabilitation programs.

The age group with the highest incidence of moyamoya disease is 0–10 years old, followed by 30–50 years old (6, 28–30). In addition, it is reported that children with unilateral often exhibit progression to typical bilateral MMD (31). In 2011, Yeon et al. (22) reported that contralateral progression was mostly observed in patients under the age of 9 years old and they developed contralateral lesions within 3 years after the initial diagnosis. Smith et al. (21) reported that a younger age at diagnosis was associated with a rapid contralateral progression. Park et al. (14) also found younger age was associated with more rapid rate of progression (age <8 years, 14.18 months and age >8, 22.38 months). Consistent with their data, our results also showed lower age was associated with progression in MMD patients. One thing worth noting, moyamoya disease is mainly divided into hemorrhagic type and ischemic type, in which children mainly present with ischemic symptoms and adult patients mainly present with hemorrhagic symptoms with high mortality (11, 32, 33). Therefore, a bias in younger age groups at greater susceptibility to progression may exist due to rapid death of patients without observed disease progression. But in any case, closer follow-up using MRI would be necessary for child patients or younger adults. Especially for children, active surgical intervention has a significant effect on restoring normal brain function before irreversible brain damage occurs.

The incidence of people with a family history of MMD are 30–40 times higher than the incidence of the general population, and patients with MMD have shown the genetic characteristics of autosomal dominant (34). Mineharu et al. found that 50% of the children of MMD patients could progress to MMD (35). Accordingly, it can be considered that patients with family history of MMD and angiographic features of MMD can be diagnosed as MMD. Our results suggested that MMD patients with family history were more likely to progress. Therefore, patients with moyamoya disease with a family history should take precautions to reduce the probability of adverse events.

Contralateral abnormality indicated equivocal or mild M1, A1, and intracranial segment of ICA stenosis on the contralateral side. We found that contralateral abnormality was a robust predictor for progression in MMD patients. Zhang et al. (23) also showed that contralateral progression survival was significantly higher in patients with contralateral abnormality than those without it. As MMD is a chronic cerebrovascular disease, some signs, especially the appearance of stenosis, are often indicative of later development. It is important to carefully assess the contralateral vessels for even very minor involvement, and if present, follow this subgroup closely.

There are some limitations. First, lack of randomized controlled studies influenced the quality of the article. Second, fewer factors that could be included in the study affected a comprehensive study of the predictors. Third, although there were two literatures with different definitions of progression, most of the articles have the same definition.

In conclusion, this meta-analysis revealed that lower age group, family history and contralateral abnormality were associated with progression in MMD patients. The same three factors are associated with the progression of unilateral MMD to bilateral. Further studies are needed to validate our results.

## Data availability statement

The original contributions presented in the study are included in the article/supplementary material, further inquiries can be directed to the corresponding author.

## Author contributions

JC and ZX completed data extraction and manuscript writing. LD completed data checking. TW completed manuscript revision. YZ and YF completed charts production. YC provided funding and guidance. All authors contributed to the article and approved the submitted version.
